# Optimization of Echo Views for Percutaneous Device Closure of Pediatric Atrial Septal Defect through the Femoral Vein Guided by Transthoracic Echocardiography without Radiation

**DOI:** 10.1155/2020/8242790

**Published:** 2020-10-31

**Authors:** Zankai Ye, Zhiqiang Li, Hanlu Yi, Yaobin Zhu, Yan Sun, Hongju Zhang, Pei Li, Ning Ma

**Affiliations:** ^1^Department of Cardiac Surgery, Beijing Children's Hospital, Capital Medical University, National Center for Children's Health, Beijing 100045, China; ^2^Department of Echocardiography, Beijing Children's Hospital, Capital Medical University, National Center for Children's Health, Beijing 100045, China

## Abstract

**Objectives:**

This study aimed to explore the selection of views for transthoracic echocardiography-guided transfemoral puncture for the device closure of pediatric atrial septal defect (ASD) without radiation.

**Methods:**

Sixty children (29 males and 31 females) were diagnosed with a central ASD, normal heart function, and no other intracardiac deformity. All procedures were performed in a surgical operating room (without radiological equipment) under basic anesthesia; the femoral vein pathway and guidance by only transthoracic echocardiography were used to complete the device closure of the ASD. The subcostal acoustic window and parasternal aorta short-axis views were used to guide the extra stiff wire and catheter into the left atrium. All procedures were performed under the subcostal biatrial section. The sheath entered the left atrium, and the apical four-chamber view was used to monitor the delivery and release of the occluder.

**Results:**

Successful closure of the ASD was achieved in all cases. The operating time from the end of the puncture to the release of the occluder was 10.36 ± 3.57 minutes. No other incisions were needed in 60 cases. No occluders were removed, and no residual shunt or pericardial effusions were detected after the procedures, during the non-ICU stay time. The average hospital stay was 2.19 ± 0.58 days.

**Conclusion:**

The accurate selection of transthoracic echocardiographic views can better ensure the safety and effectiveness of ASD closure through the femoral vein without radiation in children.

## 1. Introduction

Atrial septal defect (ASD) is a common congenital heart disease. Percutaneous interventional closure has become the main treatment for secondary ASD regardless of open-heart surgery and extracorporeal circulation [1, 2]. However, the use of radiation in traditional percutaneous interventional therapy causes radiational damage to patients and doctors and is especially damaging in children during their state of growth and development. To overcome the above shortcomings and reveal the noninvasive advantages of echocardiography, we aimed to accomplish the procedure solely under the guidance of echocardiography. The safety and efficacy of percutaneous interventional closure of ASD has been widely recognized [3–5] but has been guided by X-rays. Identifying approaches relying solely on transthoracic echocardiography to ensure the safe completion of percutaneous occlusion of ASD is important.

Thus far, few articles focused on the choices and sequence of views during the procedure. This paper aims to study the accurate selection of intraoperative-related echocardiographic views to explore and achieve the standardization of zero-radiation-guided percutaneous closure of ASD.

## 2. Materials and Methods

This study was approved by the hospital ethics committee, and informed consent was provided by the family members. Subjects: from March 1, 2018, to September 30, 2019, 60 children with central ASDs were enrolled, including 29 males and 31 females with an average age of 4.68 ± 1.27 years, an average body weight of 16.28 ± 2.8 kg, and an ASD diameter 7.58 ± 1.92 mm. Preoperative observation: the inclusion criteria were as follows: age ≥1 year; defect diameter ≥5 mm; central-type ASD with an increased right heart volume load, a distance from the margin to the superior and inferior vena cava, coronary sinus, and pulmonary veins ≥5 mm to the atrioventricular valve ≥7 mm; and septal defect with a diameter larger than the diameter of the left atrium side of the selected occluder.

The exclusion criteria were as follows: sinus ASD and primary-type ASD, endocarditis and hemorrhagic disease, thrombus in the place of the occluder, venous thrombosis at the catheter insertion, severe pulmonary hypertension leading to right-to-left shunt, and complication by other diseases requiring surgery.

### 2.1. Perioperative Observation: Operative Time and Hospital Time

All patients underwent electrocardiogram, chest X-ray, and echocardiography before the procedure, and the results were evaluated by transthoracic echocardiography during and after the procedure.

### 2.2. Postoperative Follow-Up Index

The postoperative follow-up included using electrocardiogram, chest X-ray, and echocardiography.

### 2.3. Instruments

The instruments used include Philips IE33 machine and S5-1 and S8-3 transthoracic probes.

### 2.4. Methods

**Figure 1:**
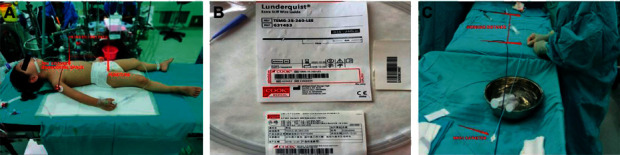
Measure the working distance: (a) the working distance is from the second intercostal space to the puncture site; (b) 0.035/260 cm extra stiff wire guide; (c) the MPA1 catheter marks the working distance.

**Figure 2:**
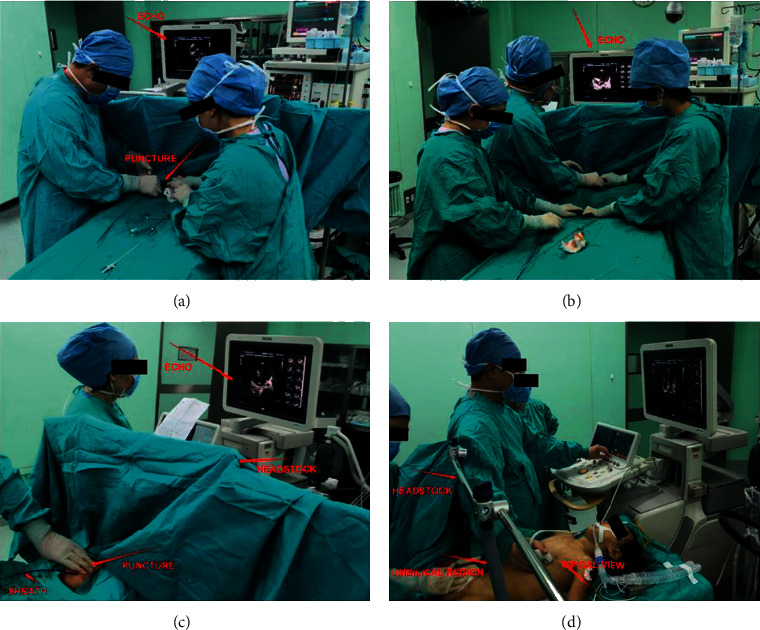
Percutaneous device closure of pediatric atrial septal defect through the femoral vein guided by transthoracic echocardiography without radiation: (a) in the operating room without radiological equipment, right femoral vein puncture point; (b, c) procedure under the guidance of transthoracic ultrasound; (d) the headstock (red arrow) and the child's umbilical region (red arrow) are vertically parallel, which can help isolate the surgical area and the ultrasound area.

**Figure 3:**
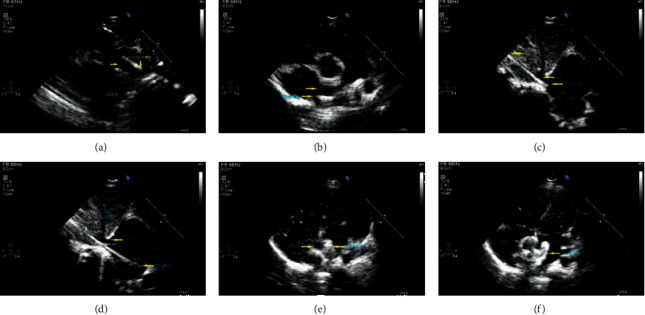
Optimization of echo views: (a) subcostal section shows the guidewire going through the inferior vena cava; (b) parasternal short-axis section of the aorta shows the catheter going through the ASD into the left atrium; (c) subcostal biatrial section shows the sheath going through the inferior vena cava; (d) subcostal biatrial section shows the sheath going through the ASD into the left atrium; (e) parasternal four-chamber view shows release of the left disc of the device; (f) apical four-chamber view shows release of the right disc of the device.

Under basic anesthesia, the children were placed in the supine position. After puncturing the right femoral vein, a 5F or 6F vascular sheath was inserted, and the 5F right heart catheter and extra stiff guidewire were sent through the vascular sheath. We completed the entire procedure under the guidance of transthoracic echocardiography (Figures 1(a)–1(c); Figures 2(a)–2(d)):

1. The subcostal view was used to guide the guidewire and catheter to search for the ASD in real time (Figure 3(a)).
2. The parasternal short-axis view of the aorta was used to guide the guidewire and catheter into the left atrium (Figure 3(b)).
3. The biatrial view of the subcostal view was applied to expose the inferior vena cava and the left and right atrium to the greatest extent possible to monitor the delivery of the sheath through the inferior vena cava through the ASD in real time; the working distance was added to withdraw the sheath and transport the internal sheath. We ensured that the delivery sheath entered the left atrium safely (Figures 3(c) and 3(d)).
4. The parasternal four-chamber view was applied to dynamically monitor the release of the occluder. First, the left atrium umbrella was released, and the delivery sheath adhering to the interatrial septum was pulled back under the real-time monitoring of transthoracic echocardiography. The right atrium umbrella was released, and the position and shape of the occluder were confirmed. Then, the delivery rod was rotated counterclockwise to release the occluder (Figures 3(e) and 3(f)). The delivery sheath was pulled out and pressed to stop bleeding, and the bandage was pressure-wrapped. A half dose of heparin (50 U/kg) was administered every 6 hours twice after the procedure.

### 2.5. Statistical Methods

The count data are expressed as numbers and percentages, and the measurement data are presented as the mean ± standard deviation. All data were analyzed using SPSS 19.0 software for statistical processing.

## 3. Results

Sixty patients underwent only transthoracic echocardiography guidance during the operation (without radiation or transesophageal echocardiography). All closures were successful, and no other incisions were changed. No thrombosis or embolism occurred, including gas embolism. There were no complications, such as bleeding at the puncture site, cardiac perforation, pericardial tamponade, or arrhythmia. There were no residual shunts in the ultrasound examinations, and no compression of the aortic valve, mitral valve leaflets, bleeding after the procedures, hemolysis, or occluder shift or fall off was observed (Table 1).

In total, 60 children were treated with a laryngeal mask. No tracheal intubation was used. After the procedure, the laryngeal mask was removed, and the patients were delivered directly to the general ward. The patients were discharged successfully the day after the procedure.

## 4. Discussion

With the widespread use of the Amplatzer occluder and the rapid development of interventional techniques, traditional radiographically guided percutaneous interventional ASD closure has been proven to be a safe and effective method [1, 2]. Some scholars [6] used intracardiac echocardiography to guide percutaneous transluminal atrial septal occlusion in the early years, but the cost of the corresponding catheter is not suitable for China's current national conditions. In recent years, to avoid radiation damage to patients and doctors, the use of echocardiography to gradually and completely replace radiation for interventional therapy has become an important method for improving traditional percutaneous interventional techniques [7]. However, how to complete the surgical procedure safely and more effectively under the guidance of transthoracic echocardiography without radiation or intracardiac echocardiography remains questionable. This manuscript aims to study the procedure guided, only by transthoracic echocardiography. The selection of the sections for the occlusion of the ASD ensures the safety and efficiency of the implementation of this new technology.

Traditional radiographically guided percutaneous septal occlusion has many merits, including no surgical scars, less pain, and shorter hospital stays [8, 9]. However, this approach requires fluoroscopy, which has adverse effects on patients and medical staff, and can cause radiation-related damage, especially in pregnant women and children, children with blood diseases, and other special populations [10]. After years of exploration, domestic and foreign experts have reported that relying on transesophageal ultrasound to close ASDs and the application of transesophageal ultrasound require tracheal intubation to prevent asphyxia, which in turn greatly increases the cost and risk of treatment. Good acoustic window conditions in children can fully guide transthoracic echocardiography. The transesophageal ultrasound was changed to transthoracic ultrasound, which avoided general anesthesia tracheal intubation, and congenital secondary ASD was treated with no incision, radiation, tracheal intubation, or extracorporeal circulation.

The 60 cases of zero-radiation pure transthoracic ultrasound-guided percutaneous ASD occlusion performed by the Department of Cardiovascular Surgery of the National Children's Medical Center did not require chest opening, avoiding X-ray irradiation and eliminating extracorporeal circulation. The 60 cases were successfully accomplished each at one time. The operation and hospitalization times of the children were significantly reduced, and no major complications occurred after the follow-up. The quality of life of the children was improved to varying degrees compared with their preoperative quality of life. Our results show that although transthoracic echocardiography is known to be less accurate than transesophageal echocardiography, transthoracic echocardiography plays an important role as the only guiding tool throughout the process, especially in pediatric patients. Many experts and scholars have demonstrated that, for a skilled operator, transthoracic ultrasound can replace transesophageal ultrasound for atrial and occlusion guidance under certain conditions [11]. Azhar reported that transthoracic ultrasound is safer than transesophageal ultrasound in the absence of atrial occlusion, leading to a significant reduction in the operating time, radiation exposure time, and application time of basic anesthesia [12]. Since echocardiography is two-dimensional, it is critical to accurately determine the position of the catheter. Under traditional radiation guidance, it is usually very easy to see the tip end and travel path of the catheter. Completing the interventional operation safely and efficiently under the guidance of only transthoracic echocardiography and the accurate selection of the ultrasound section are very important.

The author, an interventionist, confirms that surgeons are available to ensure the safety and efficiency of the interventional operation through the following operations and transformations. First, the inferior vena cava was fully exposed through the subcostal view. The interventionist inserted a multifunctional catheter and extra stiff guidewire. We can usually observe the catheter enter the right atrium through the biatrial view of the subcostal section and the rotation angle combined with the working distance [3]. The catheter was delivered into the left atrium. Simultaneously, combined with the parasternal short-axis view of the aorta, the catheter entered the left atrium. A multifunctional catheter was used to dynamically observe the travel path of the occluder delivery sheath under the subcostal view.

The anatomical path of the entire process is femoral vein-inferior vena cava-right atrium-atrial septal defect-left atrium. In the absence of X-rays, we need to achieve the entire procedure under the guidance of transthoracic echocardiography alone. Thus, it is critical to optimize the observational planes. Simple congenital heart disease with atrial septal defect is typically not associated with an abnormal inferior vena cava. Usually, complex congenital heart diseases are prone to be associated with an abnormal inferior vena cava [13]. Through subcostal echocardiographic views, we can observe the path and processing of the catheter and guidewire to the greatest extent possible. Subcostal views of children are usually easier to obtain than those of adults. The observation of the biatrial view can better reveal the relationship between the inferior vena cava and the right atrium, which is convenient for us to observe the progress and path of the catheter guidewire. In particular, the head position and movements of the delivery sheath can be displayed very clearly after we replace the delivery sheath to help us accurately locate it. The short-axis view of the aorta can clearly show the double-track sign of the delivery sheath including its head. After the position of the delivery sheath is determined, the relationship between the released device and surrounding structures can be effectively observed in combination with the four-chamber views, including the mitral valve and pulmonary veins, which ensures the safety of the device release and avoids the occurrence of complications. Furthermore, we avoid the use of harmful X-rays and expensive intracardiac ultrasound [6] to complete the entire occlusion procedure quickly and efficiently by minimizing the number of views conversions while ensuring the quality of the imaging, optimizing the process, and ensuring the quality of the procedures.

Through continuous learning and adjustment, currently, we can complete the whole process efficiently and safely without radiation. The time from the end of the puncture to the release of the occluder was only 10.36 ± 3.57 minutes, and the process could be completely performed without traditional radiation. The septal defect closure is comparable and fully demonstrates the advantages of this new technology.

This study is a single-center retrospective study, and the sample size of our group was small. In addition, the current technology has not been widely used or carried out because most current surgeries are more suitable for the completion of percutaneous ASD closure with the help of X-ray. However, we should realize the broad application of this new technology in children with congenital heart disease.

## 5. Conclusion

The accurate selection of transthoracic echocardiographic views can better ensure the safety and effectiveness of ASD closure without radiographically guided percutaneous closure.

## Figures and Tables

**Table 1:** The basic clinical situation of the 60 children.

Basic situation	ASD size (mm)	Occluder size (mm)	Actual operation time of procedures (min)
Male (29 cases)	8.24 ± 1.46	11.28 ± 2.34	7.43 ± 2.56
Female (31 cases)	6.67 ± 2.37	10.25 ± 1.57	8.31 ± 2.17

## Data Availability

The datasets used and/or analyzed during the current study are available from the corresponding author upon reasonable request.
